# Atomic‐Scale Mechanisms of Multi‐Resistance States in HfO_x_‐Based RRAM: Evolution of Atomic Electric Fields and Oxygen Vacancies

**DOI:** 10.1002/advs.202518252

**Published:** 2025-12-22

**Authors:** Wen Sun, Yuyan Wang, Ruofei Hu, Yuyao Lu, Jun Xu, Xinyi Li, Bin Gao, He Qian, Jianshi Tang, Huaqiang Wu

**Affiliations:** ^1^ School of Integrated Circuits Beijing Innovation Center for Future Chips BNRist Tsinghua University Beijing China

**Keywords:** atomic electric field, conductive filament, HfO_x_, multilevel resistive switching, oxygen vacancies, RRAM

## Abstract

The stability of multilevel resistive switching in HfO_x_‐based RRAM is crucial for enhancing matrix‐vector multiplication efficiency in computing‐in‐memory architectures, yet precise control over conductive filament formation is limited by an incomplete understanding of oxygen vacancy dynamics. Using advanced transmission electron microscopy (TEM) techniques, this study reveals key correlations among crystallographic orientation, oxygen vacancy distribution, and resistive switching mechanisms. Distinct m‐phase orientations govern resistance states: the High Resistance State (HRS) is characterized by a [101] orientation without oxygen vacancies; the Medium and Low Resistance States (MRS/LRS) exhibit a [011] orientation with selectively formed vacancies. Thermally driven m‐phase urotation ([101] ↔ [011]) facilitates oxygen vacancy migration, with vacancies preferentially occupying specific lattice sites. This alters the atomic electric fields around Hf atoms and modifies electron transport pathways. The distribution of vacancies directly controls conduction mechanisms: Schottky emission in HRS, Poole‐Frenkel emission in MRS, and Ohmic conduction in LRS, corresponding to increasing vacancy concentrations. These findings demonstrate that resistive states emerge from coupled processes: crystallographic rotation and vacancy formation reconstruct atomic electric fields, which in turn determine macroscopic conduction. This framework establishes design principles for RRAM optimization by demonstrating that precise control of thermal conductivity and voltage modulation can regulate vacancy dynamics, ensuring reliable multilevel switching.

## Graphical abstract

Atomic‐scale mechanisms governing multilevel resistive switching in HfO_x_‐based RRAM are reveal through advanced TEM. Thermally driven m‐phase rotation ([101]↔[011]) enables selective oxygen vacancy migration, which reconstructs atomic electric fields and dictates conduction—from Schottky/Poole‐Frenkel emission to Ohmic transport. This insight establishes design principles for reliable computing‐in‐memory devices.

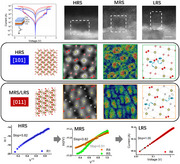

## 1 Introduction

RRAM‐based computing‐in‐memory (CIM) technology has progressed rapidly in the past decade, advancing from array‐level demonstrations to prototype chips [1, 2], and showing tremendous potential for accelerating artificial intelligence (AI) applications [3, 4]. Nevertheless, RRAM, the core device in this technology, still suffers from reliability issues, including significant variability and post‐programming conductance drift (i.e., relaxation effect) [5, 6]. When programming RRAM into multiple resistance levels for CIM applications, the overlap between different levels inevitably introduces errors in the computation of matrix‐vector multiplication (MVM), leading to considerable accuracy loss in the implementation of artificial neural networks [7, 8]. To fundamentally resolve this issue, a thorough understanding of the resistive switching mechanism of RRAM, particularly its correlation with the microstructure of conductive filament (CFs) and oxygen ion movement, is crucial [9].

A general consensus on the resistive switching mechanism in HfOx​‐based RRAM is still lacking, leaving key challenges unresolved: the microscopic mechanism of multilevel switching and the stability of resistance states. Zhang et al. reported that the CF of LRS in the Pt/HfO_x_/Pt device was composed of a core–shell structure with h‐t phase or h‐m phase, while the CF of HRS is composed of t‐phase [10]. Pan et al. reported that the amorphous HfO_x_ resistive switching layer in the W/HfO_x_/HfO_y_ (30 nm)/Pt device transformed into a mixed composition of m phase and o phase after repeated cycling operations [11]. These results indicate that the CF in HfO_x_ RRAM adopts specific crystal structures within the Hf–O phase diagram. Since there are more than 13 metastable phases in the Hf‐O system [12], HfO_x_‐based RRAM undergoes complex physical and chemical processes during resistive switching [13]. This necessitates comprehensive research in three areas: device structure, operation scheme, and microscopic characterization. Previous research has mainly examined the effects of device structure and operation scheme on the electrical properties of HfO_x_‐based RRAM [14], whereas microscopic characterization has mostly addressed the conduction mechanisms of HRS and LRS, often neglecting MRS [15]. In addition, studies on the microstructure of CFs have been limited to analyses of CF morphology and composition, with little investigation into the relationship between microstructure and device conductance [16].

To address these questions, we systematically characterized the microstructure of the CFs in a cycled TiN/TaO_x_​/HfO_x_​/TiN RRAM device and analyzed the conduction mechanisms underlying its three distinct resistance states (HRS, MRS, and LRS). The crystal structure of the CFs in these states and the associated changes in oxygen concentration were characterized, and the morphological evolution of the CFs under heating was examined using in situ TEM and DPC. Based on these experimental data, we investigated the migration of oxygen ions in the CFs, the formation of oxygen vacancies driven by the electric field, and crystallographic reorientation of the CF upon heating. The change in the distribution of oxygen vacancies was analyzed using electron energy loss spectroscopy (EELS). Compared with the previous studies that primarily investigated phase evolution, our work reveals a previously underappreciated coupling among crystallographic reorientation, oxygen vacancy distribution, and atomic‐scale electric field reconstruction. We propose a “crystal phase rotation–atomic electric field–multistate coupling” mechanism, fundamentally governed by thermally induced m‐phase rotation. This mechanism offers a deeper and more comprehensive framework for understanding and designing multilevel RRAM devices.

## 2 Results and Discussion

### 2.1 TEM Characterization of Multi‐Resistance States

**FIGURE 1:**
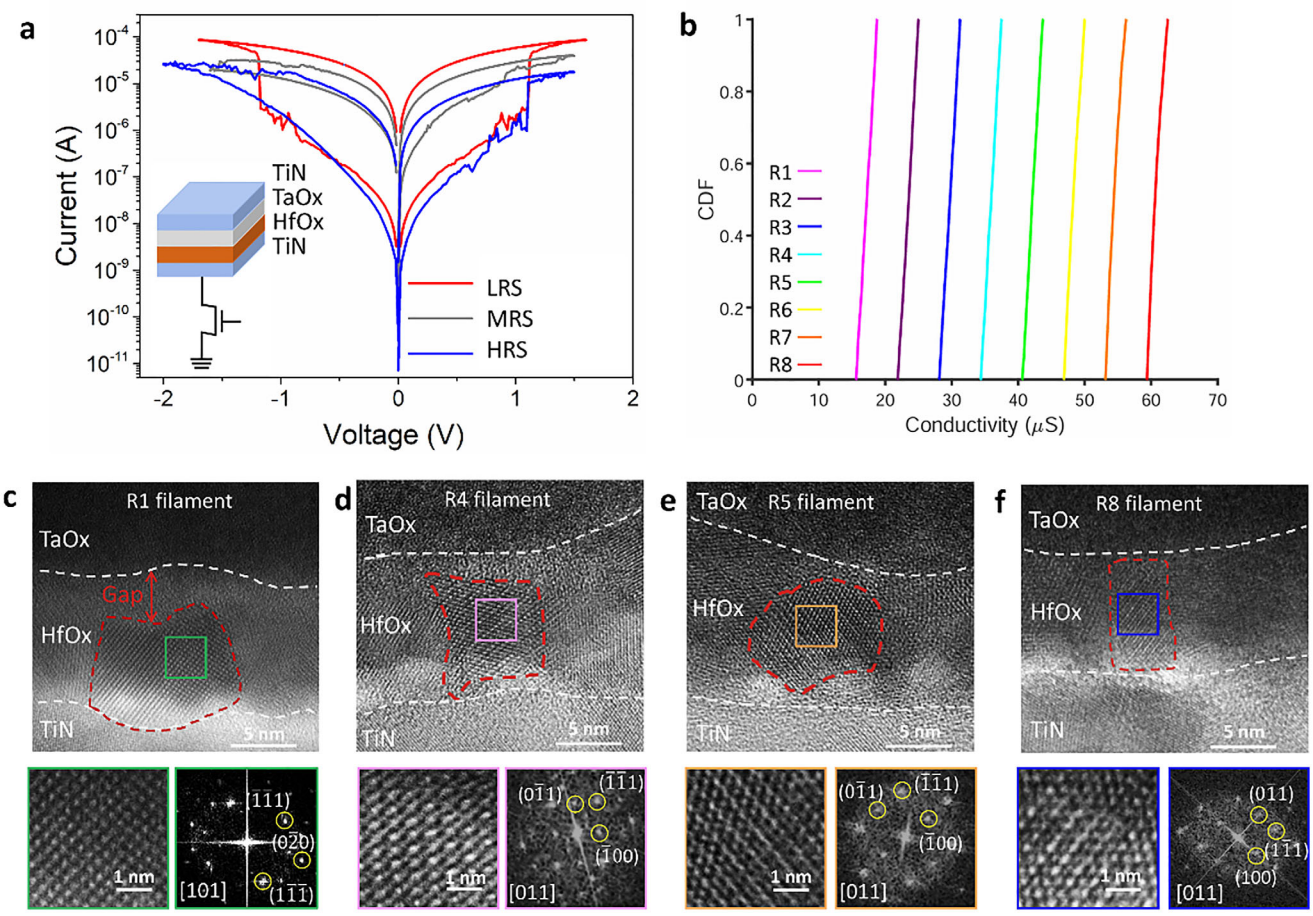
Crystal orientation change of HfO_x_ observed in multilevel resistance states. (a) Current‐voltage (I–V) curves from cycling operations comparing LRS, MRS, and HRS. Data are presented as representative curves from individual devices. (b) The cumulative distribution function (CDF) plot shows the programming of a 1K RRAM array into eight distinct conductance states (*n* = 1,024 devices). Statistical data were analyzed using descriptive statistics and CDF analysis (Origin software); no formal hypothesis testing was applied. (c–f) HR‐TEM images of conduction filaments. Enlarged images and corresponding electron diffraction patterns are shown for the HRS device R1 (c, *n* = 7 for HRS), the MRS device R4 (d, *n* = 7 for MRS) and R5 (e), and the LRS device R8 (f, *n* = 9 for LRS). All microstructural features were reproducibly observed across the tested samples. Scale bars: 5 nm in (c)–(f); 1 nm in enlarged images. No significance symbols are used; all results are qualitative and representative.

Electrical operations were performed on 128 × 8 devices within a 1K array, with each column programmed to a different resistance state. Microstructural characterization was then conducted on these eight columns. Figure 1a presents the I–V curves of three representative devices, including HRS, MRS, and LRS devices. Pulse‐controlled RRAM operations achieved eight resistance states within the 1K array, as illustrated in Figure 1b. HR‐TEM characterization of typical devices revealed that the HfO_x_ crystalline structure of HRS device R1 exhibited a 2–3 nm gap at the interface with the TaO_x_ layer, corresponding to the rupture point of the conductive filament (CF) (Figure 1c). In contrast, MRS devices R4 and R5 displayed rectangular prism‐shaped HfO_x_ crystalline structures bridging the TaOx layer and TiN bottom electrode (Figure 1d,e). The LRS device R8 exhibited a similar rectangular prism‐shaped HfO_x_ crystalline morphology (Figure 1f). Phase identification of conductive filaments based on high‐resolution imaging demonstrated that all four resistance states possessed monoclinic (m‐phase) crystal structures (Tables S1–S3). Notably, the HRS device exhibited an m‐phase orientation along the [101] direction, whereas MRS and LRS devices exhibited [011]‐oriented m‐phase structures. Further TEM analysis is presented in Figures S1 and S2.

**FIGURE 2:**
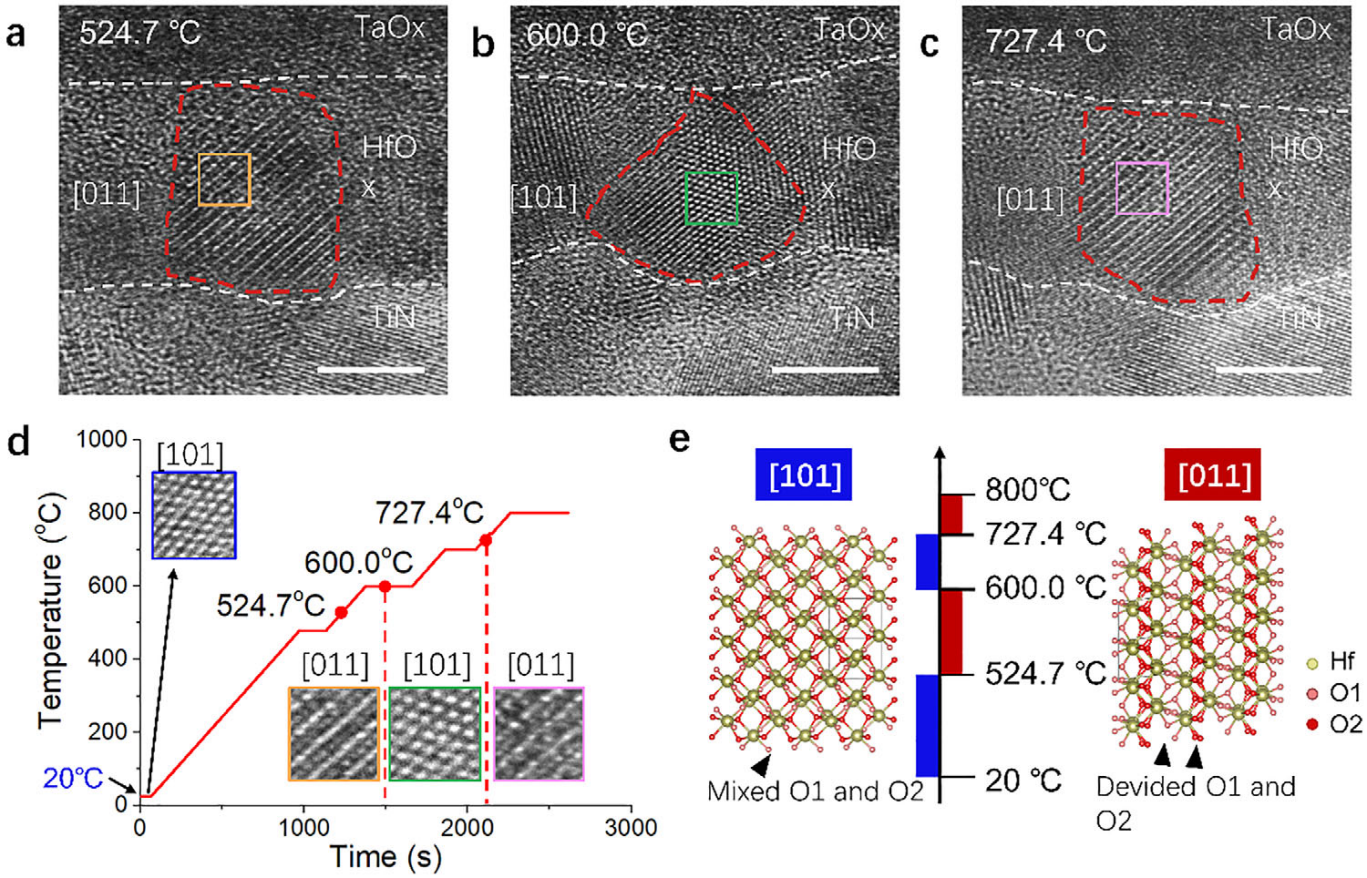
Crystal orientation change of HfO_x_ observed through in situ heating TEM. (a–c) HR‐TEM images showing m‐phase orientation at 524.7, 600.0, and 724.4°C, respectively; sample size *n* = 4 (representative in situ heating sequence). (d) Time‐temperature curve of the in situ heating process, with insets showing enlarged high‐resolution TEM images for each heating stage. (e) Atomic model illustrating m‐phase reorientation during heating. Data presentation is qualitative; no statistical significance testing was performed due to the nature of in situ observation. Scale bars: 5 nm in TEM images.

We performed in situ heating experiments with atomic‐scale resolution on HfO_x_‐based RRAM devices to explore the impact of Joule heating on crystalline phase evolution, revealing a thermally induced rotational transition of the m‐phase (Figure 2). At room temperature, the m‐phase was initially oriented along the [101] direction. Upon heating to 524.7 °C, the crystal orientation rotated to [011], which reverted to [101] at 600.0 °C. Further heating to 724.4 °C induced another rotation to the [011] orientation (Figure 2a–d). The phase transition initiated from the crystal edges and gradually propagated throughout the entire crystalline region, with continuous structural evolution observed during heating and stabilization.

The correlation between programmed resistance states and crystalline phase configurations, combined with in situ observations of thermal phase evolution, demonstrates that temperature facilitates resistive switching in HfO_x_‐based RRAM by inducing thermally activated lattice reorientation between [101] and [011] m‐phase configurations. In the m‐phase HfO_2_ crystal structure, Hf⁴⁺ ions coordinate with seven O^2−^ atoms, forming a distorted of corner‐ and edge‐sharing HfO_7_ pentagonal bipyramids. Two distinct oxygen sub‐lattices are identified: (1) O1 sites, where O^2−^ ions are bonded to three equivalent Hf⁴⁺ cations in a distorted triangular noncoplanar geometry, and (2) O2 sites, where O^2−^ ions are tetrahedrally coordinated with four equivalent Hf⁴⁺ cations, constituting distorted corner‐ and edge‐sharing OHf_4_ tetrahedra [17]. In temperature ranges from room temperature to 524.7 °C and from 600.0 to 724.4 °C, the [101]‐oriented m‐phase dominates, exhibiting uniformly distributed three‐ and four‐fold coordinated oxygen atoms that enhance oxygen bond stability (Figure 2e). This atomic configuration suppresses ion migration and maintains ruptured conductive filaments, which corresponds to the high resistance state. Conversely, temperature ranges of 524.7–600.0 °C and above 724.4 °C, the [011] orientation prevails, characterized by alternating layers of three‐fold coordinated oxygen planes interleaved with Hf atomic sheets. This layered architecture creates two synergistic effects critical for low‐resistance operation: it generates low‐energy oxygen vacancy sites (O1 sites) and establishes continuous ion migration channels between electrodes, thereby enabling conductive filament reconstruction for MRS and LRS devices.

It is important to note that the in situ heating apparatus employed in this study applies uniform thermal excitation across the entire device structure. This condition is fundamentally distinct from the actual operational state of the conductive filament within a working device. Three key differences arise during real operation. First, Joule heating generated by electrical current is highly localized along the current path defined by the conductive filament itself, rather than being uniformly distributed. Second, the inherent disparity in thermal conductivity between the materials forming the top and bottom interfaces of the device further contributes to nonuniform heat distribution within the filament region. Furthermore, the actual morphological evolution of the conductive filament is governed not solely by thermal effects but also critically influenced by the distribution of the applied electric field. This concurrent electric field interaction leads to significant differences in the filament's evolution under real operating conditions, such as the formation of oxygen vacancies, their migration pathways, and the stability of specific crystalline phases. Nevertheless, the temperature range explored in our in situ experiments (room temperature to 724.4 °C) is highly representative of the true operating conditions experienced by conductive filaments during resistive switching, which multiple studies report to be between 277 and 1300 °C during switching [18, 19].

### 2.2 Evolution of Atomic Electric Fields and Oxygen Vacancies

To elucidate the influence of oxygen atoms and vacancies on the electrical properties of m‐phase HfO_x_, we employed the DPC‐STEM technique to achieve simultaneous atomic‐scale mapping of oxygen positions and electric field distributions. The integrated differential phase contrast (iDPC) technique provides sub‐nanometer resolution for precise localization of oxygen sub‐lattices (O1 three‐fold and O2 four‐fold coordinated sites), while DPC quantifies atomic‐scale electric fields through probe center‐of‐mass (CoM) displacement measurements [20]. This combined approach is validated as demonstrated in studies of GaN [21] and Au single‐atom systems [22]; its reliability was further confirmed by Avizo‐based data correlation analysis. To observe the formation sites of oxygen vacancies at the atomic scale, we conducted observations along the [010] zone axis across multiple resistance states of the m‐phase. This orientation enables precise identification of oxygen vacancy locations due to the alternating arrangement of O1 and O2 sites. Additionally, the [011] zone axis was chosen as a comparative orientation for observation. Additionally, the [011] zone axis was selected for comparison; in this orientation, oxygen atoms at O1 and O2 sites exhibit a uniform distribution, enabling clearer analysis of atomic‐scale electric field variations between different resistance states. This dual‐axis approach supports the correlation between oxygen vacancies and electric fields, and provides a robust framework linking microscopic mechanisms to macroscopic device characteristics.

**FIGURE 3:**
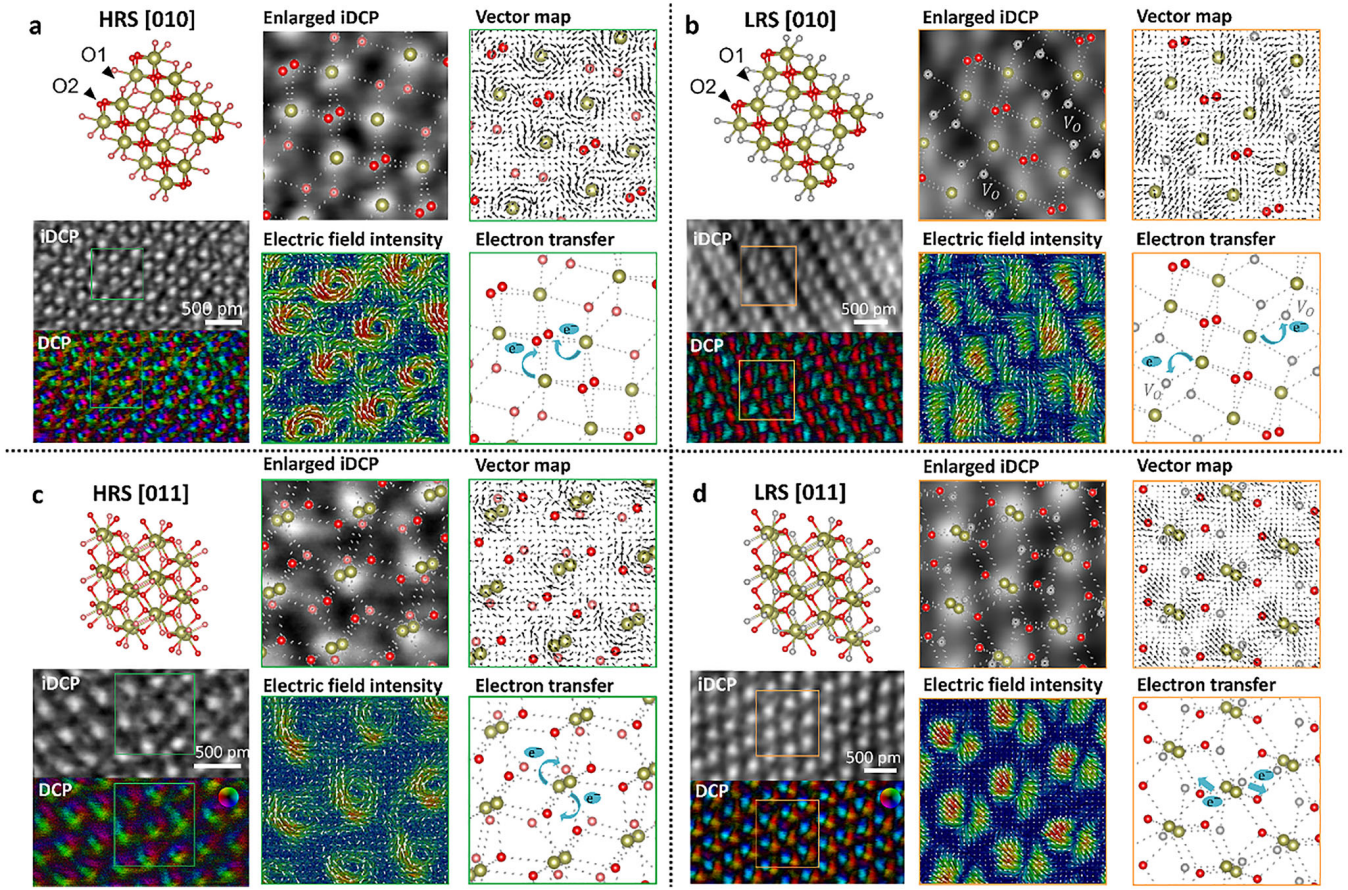
DPC characterization of oxygen vacancies and atomic‐scale electric fields within the m‐phase. Panels (a, b): [010] zone axis observations for HRS (*n* = 7 devices) and LRS (*n* = 9 devices). Panels (c, d): [011] zone axis observations for HRS (*n* = 7) and LRS (*n* = 9). Each set includes atomic models, iDPC and DPC images, enlarged overlays, electric field intensity maps, and electron pathway vector maps. Microstructural and field mapping results are representative and reproducible across the sample sets; data presentation is qualitative, without hypothesis testing or significance symbols.

Figure 3 presents our atomic‐scale electric field characterization results for both HRS (R1) and LRS (R8) devices along the [010] and [011] zone axes, obtained using the DPC technique. The iDPC magnified view with atomic model overlay in Figure 3a reveals complete occupancy at both O1 (three‐fold coordinated) and O2 (four‐fold coordinated) oxygen sites in HRS devices, indicating minimal oxygen vacancy formation. Avizo‐processed DPC analysis yields electric field intensity maps and vector plots, demonstrating convergent field distributions centered around Hf atomic columns in HRS devices. The field vectors orient from Hf⁴⁺ cations toward O2 sites, suggesting electron confinement within OHf_4_ tetrahedra that requires substantial energy to overcome the potential barrier [23], consistent with the observed low macroscopic conductivity.

In striking contrast, LRS (R8) devices exhibit fundamentally distinct characteristics. Figure 3b presents DPC analysis of [010]‐oriented m‐phase in LRS devices. The iDPC‐atomic model overlay identifies selective oxygen vacancy formation at O1 sites while maintaining O2 site integrity. Corresponding field intensity maps show divergent field distributions around Hf atoms, with field maxima shifting toward vacancy sites. Field vector analysis reveals preferential electron migration pathways from Hf atoms to oxygen vacancies (V_O_) [24], indicating defect‐assisted hopping conduction through percolation paths formed by V_O_ clusters. This microscopic mechanism accounts for the enhanced macroscopic conductivity in LRS devices.

Consistent with [010]‐oriented DPC observations, [011]‐oriented m‐phase in HRS devices exhibits convergent electric field distributions where atomic electric field vectors form concentric patterns around Hf atoms, directing electron migration from Hf cations to oxygen anions (Figure 3c). The LRS devices demonstrate vacancy‐induced field reconstruction in the [011] orientation as well (Figure 3d). Oxygen vacancies preferentially form at O1 sites, inducing divergent field distributions with enhanced intensity near vacancy sites. Atomic electric field vectors reorient from Hf atoms toward neighboring oxygen vacancies (V_O_), establishing electron conduction pathways along Hf→V_O_ directions.

Comparative analysis of [010] and [011] orientation of the m phase reveals the formation principles of oxygen vacancies and their regulatory effects on the atomic electric fields of Hf atoms. The formation of oxygen vacancies demonstrates site selectivity: vacancies preferentially nucleate at O1 sites, which is consistent with first‐principles calculations demonstrating that the formation energy of O1‐site vacancies (2.1 eV) is significantly lower than that of O2‐site vacancies (3.4 eV) [25]. The fundamental mechanism of oxygen vacancy regulation of HfO_x_ electrical properties lies in atomic‐scale electric field reconstruction that modifies electron transition pathways [26]. In the absence of oxygen vacancies, Hf atoms exhibit convergent electric field distributions that confine electrons around the Hf sites, requiring thermal excitation to overcome the potential barrier—a configuration macroscopically manifested as the high resistance state (HRS). Upon oxygen vacancy formation, the Hf atomic electric fields transform into divergent distributions, where vacancies serve as shallow traps that reduce potential barriers, enabling electron conduction through tunneling or thermally‐assisted transitions—a configuration corresponding to the low resistance state (LRS).

**FIGURE 4:**
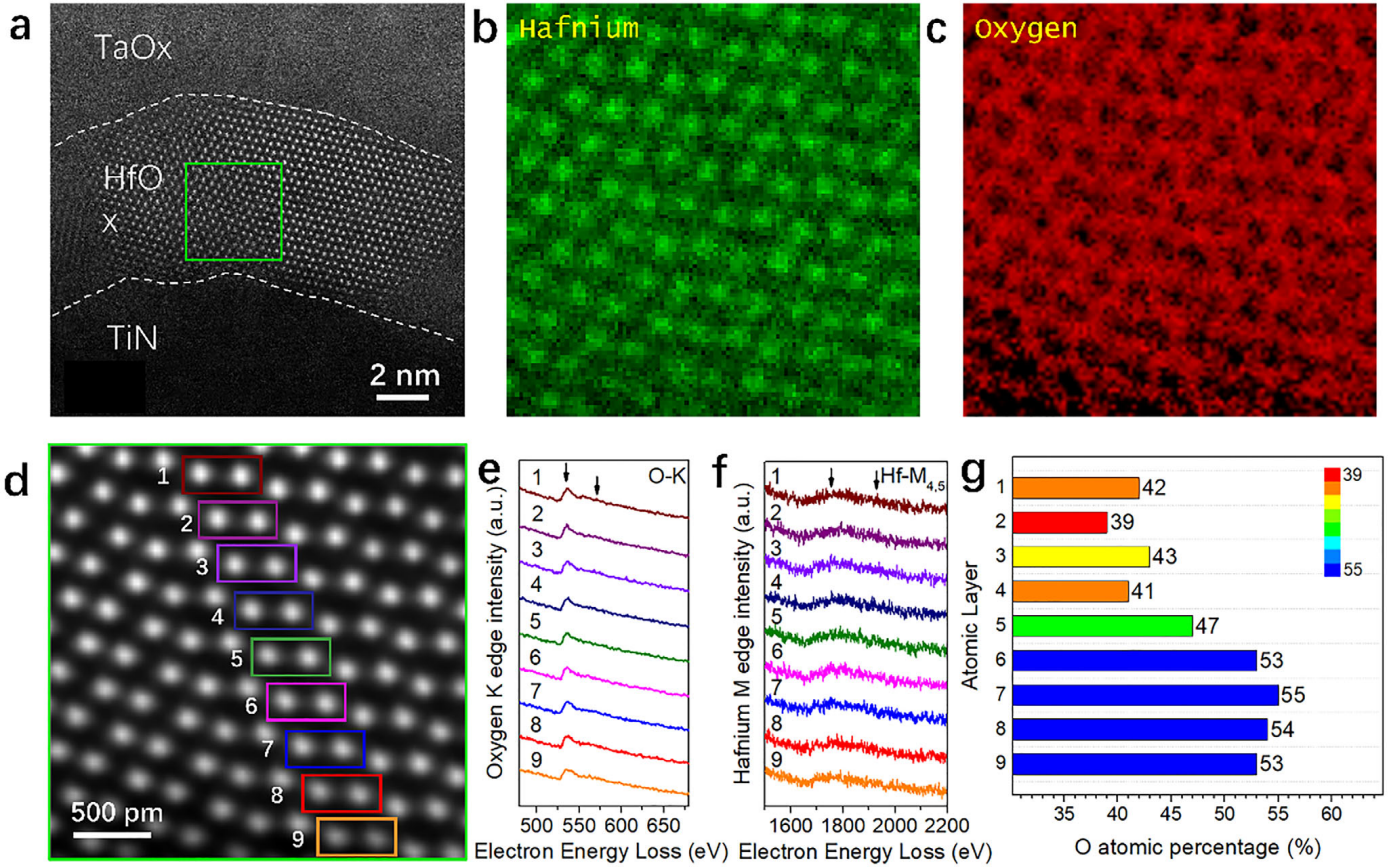
Atomic‐resolution EELS compositional mapping and analysis. (a) Illustration of the EELS sampling region. (b) Atomic mapping of Hf elements. (c) Atomic mapping of O elements. (d) ADF image of the EELS acquisition region, with color‐coded boxes indicating spectral extraction areas. (e, f) EELS spectra of O and Hf elements extracted from the color‐coded regions. (g) Quantitative analysis of oxygen content percentage variations across regions using EELS spectra. Sample size for device states: HRS (*n* = 7), MRS (*n* = 7), LRS (*n* = 9). Data presentation is based on representative mapping and spectra from each resistance state; no formal statistical tests or significance symbols were applied.

To determine the spatial distribution of oxygen vacancies, we employed electron energy‐loss spectroscopy (EELS) in scanning transmission electron microscopy (STEM) to analyze the atomic‐scale lattice of the HfO_x_ layer in LRS (R8) devices. The energy acquisition range was set at 0–2200 eV, encompassing both the O‐K edge and Hf‐M_4,5_ edges [27], enabling simultaneous quantification of O: Hf atomic ratios and oxygen vacancy distributions from a single spectral dataset. Figure 4a illustrates the measurement region within the device stack for [110]‐oriented m‐phase analysis. Atomic‐resolution EELS mapping yielded distinct atomic distributions for Hf (Figure 4b) and O (Figure 4c), while the corresponding annular dark‐field (ADF) image (Figure 4d) revealed nine Hf atomic layers. Quantitative analysis was performed on paired Hf atomic regions (color‐coded boxes in Figure 4d), with representative O‐K edge and Hf‐M4,5 edge spectra shown in Figure 4e,f, respectively. Quantitative EELS analysis (see Methods for details) revealed that the oxygen atomic percentage ranges from 39 ± 3 % to 55 ± 3 % (Figure 4g), significantly lower than the stoichiometric HfO_2_ value of 66.6%. This corresponds to oxygen vacancy concentrations ranging from 14.4 ± 3 % to 41.4 ± 3 % (calculated as described in the Methods section), consistent with the oxygen deficiency observed via iDPC.

The spatial distribution of oxygen atomic percentages reveals atomic‐scale nonuniformity in oxygen vacancy distribution, potentially associated with local lattice distortions or defect clustering. We observe a clear gradient where oxygen content progressively increases from the HfO_x_/TaO_x_ top interface toward the TiN/HfO_x_ bottom interface (Figure 4g), indicating a corresponding decrease in oxygen vacancy concentration. This phenomenon may originate from the lower oxygen binding energy of Ti atoms compared to Hf atoms [28], facilitating preferential oxygen extraction from HfO_x_ and resulting in a chemical gradient.

The atomic‐scale heterogeneity and macroscopic gradient of oxygen vacancies significantly influence the electrical properties of LRS (R8) devices. Regions with higher vacancy concentrations (particularly near the HfO_x_/TaO_x_ interface) likely act as sources of increased charge carrier density. Under applied electric fields, these carriers migrate along the oxygen vacancy gradient toward the TiN/HfO_x_ interface, establishing localized conduction pathways that collectively reduce the macroscopic device resistance. Further compositional analysis is presented in Figure S3.

### 2.3 Conduction Mechanism and Switching Mechanism

Building upon these characterization results (from Figures 1, 2, 3, 4), we establish a comprehensive physical model elucidating the conduction and resistive switching mechanisms in multilevel RRAM devices. The conduction mechanisms of the multilevel RRAM devices, including the high resistance state, medium resistance state, and low resistance state, are clarified by fitting their current‐voltage (I–V) characteristics with distinct transport models, thereby revealing the electron transport laws within the band structures. By combining in situ heating characterization results, we deduced the operational temperature ranges of the conductive crystals under the three resistance states. We also clarified the driving effect of Joule heat on the evolution of crystal structures. Based on the TEM and atomic‐resolution characterization results of the conductive crystals, we established the distribution models of oxygen vacancies within the conductive filaments. Multidimensional analyses enable a systematic and detailed understanding of the conduction mechanisms in multilevel RRAM.

#### 2.3.1 High Resistance State

**FIGURE 5:**
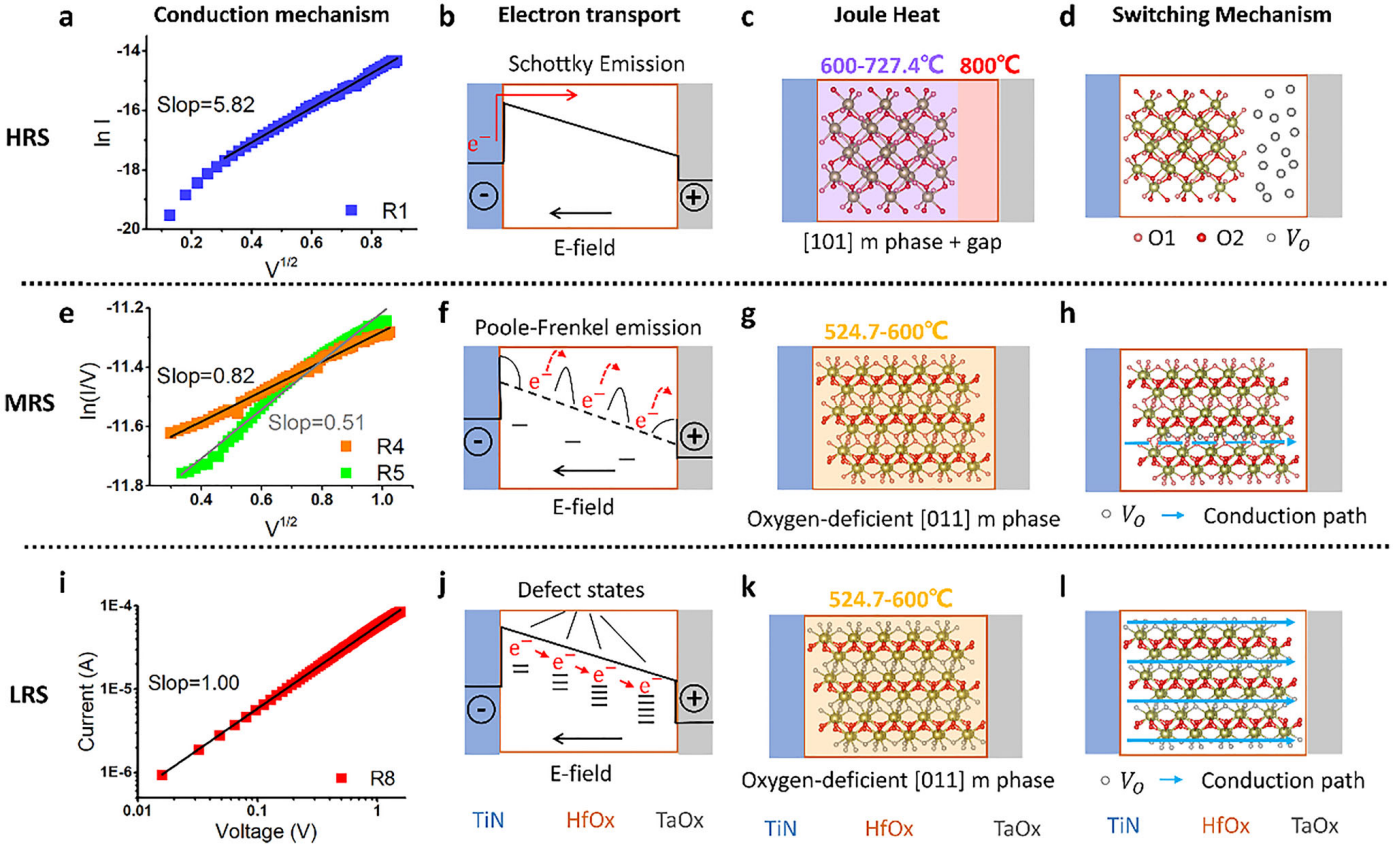
Conduction and switching mechanisms. (a) I–V curve of the HRS device, showing a linear ln(I)‐*V* ^1/2^relationship, confirming the Schottky emission mechanism (*n* = 7 for HRS). (b) Electron transport band structure for the HRS CF. (c) Joule heating effect on reorientation in the HRS CF. (d) Microstructure of the HRS CF. (e) I–V curve of the MRS device, exhibiting a linear ln(I/V)‐*V* ^1/2^ relationship consistent with Poole‐Frenkel conduction (*n* = 7 for MRS). (f) Electron transport band structure of the MRS CF. (g) Joule heating effect on reorientation in the MRS CF. (h) Microstructure of the MRS CF. (i) Double‐logarithmic I–V curve of the LRS device, showing a slope of 1, in accordance with Ohmic conduction (*n* = 9 for LRS). (j) Electron transport band structure of the LRS CF. (k) Joule heating effect on reorientation in the LRS CF. (l) Microstructure of the LRS CF. Data are presented as representative curves and images; no statistical significance testing was performed, and no significance symbols are used. Mechanism assignments are based on qualitative physical analysis.

For HRS devices, the I–V characteristics in the intermediate voltage range (0.09–0.81 V) exhibit a linear fitting relationship between lnI‐V^1/2^ (Figure 5a) with a slope of 5.82, which is consistent with the theoretical signature of Schottky Emission [29]. This confirms that the dominant conduction mechanism involves the transport of thermally excited electrons overcoming the potential barrier at the metal‐semiconductor interface. This behavior originates from the [101]‐oriented m‐phase of the conductive filament present in HRS devices, which contains negligible oxygen vacancies. Atomic‐scale characterization reveals a convergent state of the electric field around Hf atoms, which strongly confines electrons and induces a high Schottky barrier at the TiN/HfO_x_ m‐phase interface (Figure 5b). Consequently, electron transport requires thermal excitation energy provided by Joule heating to surmount this barrier and migrate toward the HfO_x_/TaO_x_ interface, aligning with barrier‐dominated transport behavior.

During the Reset operation, Joule heating serves as the pivotal driver for CF structural transformation. This heating primarily arises from current‐induced self‐heating, where the Joule heating power scales quadratically with current. Monte Carlo simulations in the literature indicate localized temperatures reaching 700–800 °C [30]. The thermal conductivity mismatch between stacked materials exacerbates heat localization at the HfO_x_/TaO_x_ interface, because TaO_x_ (0.9–4 W/(m·K)) exhibits significantly lower thermal conductivity than TiN (29.1 W/(m·K)) [31], impeding heat dissipation and promoting interfacial heat accumulation. In situ heating experiments (Figure 2b) demonstrate the stability of the m‐phase at 600.0–724.4 °C. We postulate that Joule heating during the Reset operation elevates the interface temperature beyond 724.4 °C (up to 800 °C), inducing melting of the m‐phase lattice at the HfO_x_/TaO_x_ interface. This results in amorphization and gap formation (Figure 5c). Additionally, positively charged oxygen vacancies within the m‐phase migrate toward the HfO_x_/TaO_x_ interface under negative bias and accumulate in the gap region, collectively disrupting conduction continuity (Figure 5d). Thus, the physical disconnection of the conductive channel constitutes the fundamental origin of HRS. This involves two synchronous processes: (1) formation of a Schottky barrier by the oxygen‐vacancy‐deficient residual m‐phase crystal, and (2) melting‐induced amorphization of the conductive filament at the HfO_x_/TaO_x_ interface, generating a structural gap. These dual structural modifications together result in a substantial increase in resistance.

#### 2.3.2 Medium Resistance State

The MRS devices exhibit a linear ln(I/V)‐V^1/2^ relationship (Figure 5e) with slopes of 0.82 and 0.51, characteristic of the Poole‐Frenkel (P‐F) emission conduction mechanism [16, 32]. This indicates that the dominant conduction mechanism involves the thermal excitation of electrons from crystal traps (oxygen vacancies). The excited electrons then hop between traps with field assistance, during which the applied field reduces the Coulombic potential barriers (Figure 5f). This behavior originates from the [011]‐oriented m‐phase crystal structure of the MRS conductive filament, which bridges the electrodes and contains a limited number of selectively formed oxygen vacancies at O1 sites. These oxygen vacancies create localized defect energy levels in the band structure, fundamentally distinct from the high Schottky barrier in HRS. Trap energy levels and electric‐field‐assisted barrier reduction [33] in this band configuration collectively enable a markedly lower electron transport barrier compared to HRS.

The formation of MRS during the Set process is driven by Joule heating. The localized temperature induced by current self‐heating operates within the range of 524.7–600.0 °C. In situ characterization confirms that the [011]‐oriented m‐phase crystal maintains structural stability within this temperature regime. Joule heating enables the detachment of oxygen atoms from O1 sites, generating a low concentration of oxygen vacancies (Figure 5g). The low thermal conductivity of TaO_x_ exacerbates heat accumulation near the HfO_x_/TaO_x_ interface, promoting the localized formation of oxygen vacancies in this region. These vacancies act as traps that facilitate electron‐hopping transport, creating a localized trap‐assisted conduction path. Atomic‐scale electric field characterization reveals that oxygen vacancies induce local divergence of the electric field around adjacent Hf atoms, thereby favoring electron migration. However, the insufficient density of vacancies and absence of continuous conduction pathways restrict current flow, maintaining resistance at an intermediate level. Thus, the resistive switching in MRS fundamentally arises from the localized formation of oxygen vacancies within the m‐phase (Figure 5h).

#### 2.3.3 Low Resistance State

The I–V characteristics of LRS devices exhibit strictly linear behavior (Figure 5i), confirming Ohmic conduction. This indicates negligible potential barriers during electron transport, enabling continuous migration [34]. The mechanism originates from the [011]‐oriented m‐phase of its conductive filament, where oxygen vacancies at O1 sites display a gradient distribution (Figure 4g). This gradient establishes continuous defect energy levels in the band structure, permitting free electron migration between the conduction band and defect levels (Figure 5j). Atomic‐scale electric field characterization reveals a “divergent state” around Hf atoms, facilitating efficient electron migration from Hf atoms toward oxygen vacancies. This is distinct from the conduction mechanisms observed in HRS and MRS.

The formation of the LRS during the Set process is similarly driven by Joule heating within the same temperature range as the MRS (524.7–600.0 °C, Figure 5k). However, higher currents or longer heating durations substantially increase oxygen vacancy concentrations. The combined effects of Joule heating (current self‐heating) and the relatively low thermal conductivity of the TaO_x_ layer induce interfacial heat accumulation. This thermal localization drives massive oxygen vacancy generation near the TaO_x_ interface, establishing the critical gradient distribution. This gradient configuration directly transforms conduction pathways from localized trap chains to fully interconnected, continuous oxygen vacancy chains (Figure 5l). The resulting multi‐path oxygen vacancy chain structure enables electron migration with minimal barriers, constituting the fundamental microscopic origin of the LRS.

The resistive switching behavior of multilevel RRAM fundamentally originates from the dynamic evolution of oxygen vacancies in conductive filaments: the HRS is dominated by disconnected vacancy‐free m phase, where electron transport requires overcoming Schottky barriers (Figure 5a–d); the MRS relies on local oxygen vacancy traps for Poole‐Frenkel hopping (Figure 5e–h); and the LRS is determined by continuous oxygen vacancy chains enabling Ohmic transport (Figure 5i–l). Joule heat regulates the formation, migration, and distribution of oxygen vacancies through control of the thermal environment, ultimately enabling nonvolatile storage of multilevel states. This physical model integrates multidimensional data from I‐V fitting, in situ heating, and atomic‐scale characterization, providing a theoretical foundation for performance optimization of multilevel RRAM.

Our study introduces a fundamentally new mechanistic framework for HfO_x_‐based RRAM by directly linking crystal lattice rotation, vacancy migration pathways, and atomic‐scale electric field reconstruction. Compared with previous works that focus on filament morphology or vacancy channel formation, we reveal that crystallographic reorientation acts as the precursor event governing oxygen vacancy mobility and subsequent electric field modulation, thereby dictating macroscopic conduction behavior. This comprehensive “crystal reorientation–vacancy dynamics–electric field reconstruction” mechanism not only integrates key insights from earlier models [10, 11], but also extends beyond them by demonstrating how resistive switching is fundamentally controlled at the atomic scale. Our findings are applicable to HfO_x_‐based RRAM devices because the monoclinic phase is a thermodynamically stable crystal structure, and the formation and migration of oxygen vacancies play a decisive role in the resistance switching behavior of RRAM. However, we acknowledge that variations in electrode materials, oxide stoichiometry, and operating conditions can influence the manifestation of these mechanisms, and alternative filament phases may lead to different conduction pathways in certain device architectures.

## 3 Conclusions

This work establishes the “crystal phase–vacancy–electric field tri‐level regulation mechanism” as a unifying design principle for multilevel resistive switching in HfOx‐based RRAM. We demonstrate that resistance state transitions originate from thermally driven crystal rotation, which creates migration channels for oxygen vacancies. Critically, oxygen vacancy formation directly reconstructs local atomic electric fields: in the absence of vacancies, Hf atoms exhibit convergent electric fields that confine electrons and establish high Schottky barriers, whereas vacancy formation induces a transition to divergent electric fields that substantially reduce electron migration barriers. Consequently, this atomic‐scale field evolution governs macroscopic conduction mechanisms, where the gradient distribution of oxygen vacancies progressively transforms electron transport pathways. Electron transport progressively evolves from thermally activated Schottky emission in the vacancy‐free high‐resistance state (HRS), through electric‐field‐assisted trap hopping governed by sparse vacancies (Poole‐Frenkel emission) in the medium‐resistance state (MRS), to barrier‐free Ohmic conduction enabled by continuous vacancy chains in the low‐resistance state (LRS). This study reveals that resistance states fundamentally constitute a cascaded process where crystal reorientation promotes oxygen vacancy formation, thereby reconstructing atomic electric fields, which ultimately govern electronic band structures through vacancy distribution patterning.

To enhance device performance stability, optimization strategies should focus on three key aspects: thermal management, defect engineering, and interface modification. First, incorporating high‐thermal‐conductivity interfacial layers, such as aluminum nitride (AlN), can homogenize heat distribution, while dynamic pulse operation protocols may precisely control Joule heating zones to prevent excessive amorphization or oxygen vacancy aggregation. Second, strategic doping, such as yttrium (Y) or aluminum (Al), can stabilize oxygen vacancy formation energies to suppress stochastic defect fluctuations, coupled with crystal orientation optimization to improve thermal stability. Finally, implementing buffer layers at TaO_x_/HfO_x_ interfaces can reduce Schottky barriers and mitigate thermal accumulation in HRS. The synergistic implementation of these measures would significantly improve resistance state uniformity and endurance, providing critical technical support for transformative applications in high‐density multilevel storage and neuromorphic computing. Future research should focus on validating the universality and scalability of the tri‐level mechanism in vertically integrated 3D crossbar arrays, as well as exploring the real‐time dynamics of crystal phase, vacancy, and electric field evolution to emulate synaptic plasticity and learning behaviors.

## 4 Methods

### 4.1 Device Fabrication

Metal‐oxide‐semiconductor field‐effect‐transistor (MOSFET) circuits were constructed using a standard CMOS foundry with a technology node of 130 nm. An HfO_x_ RRAM was integrated at the transistor's drain (1T1R structure) using a TiN/TaO_x_/HfO_x_/TiN stack. The HfO_x_ layer was deposited on the TiN bottom electrode via atomic layer deposition (ALD). The thickness was determined to be 8.4 ± 0.2 nm by cross‐sectional TEM statistical analysis of over five different devices. The TaO_x_ oxygen reservoir layer was deposited through magnetron sputtering, and the thickness was determined to be 42.2 ± 0.3 nm.

### 4.2 Electrical Characterization

The electrical characteristics of the HfO_x_ RRAM were measured at room temperature on a probe station interfaced with an Agilent B1500A Semiconductor Device Analyzer operating in DC sweep mode. The eight resistance states were programmed into the 1K RRAM array using a pulse‐based write‐verify scheme, resulting in the cumulative distribution shown in Figure 1b. The resistance of the first‐row device corresponds to the R1 device, the second‐row device to the R2 device, and so on, defining the device resistance levels for R3 through R8. From these eight resistance levels, three representative states were selected for comprehensive analysis of microstructure and conduction mechanisms: the high‐resistance state (HRS) device was the R1 device with the highest resistance, the low‐resistance state (LRS) device was the R8 device with the lowest resistance, and the medium‐resistance state (MRS) was represented by the R4 and R5 devices, whose resistance values lie between those of the R1 and R8 devices.

### 4.3 TEM Characterization

Lamella specimens of the TiN/TaO_x_/HfO_x_/TiN RRAM for transmission electron microscopy (TEM) characterization were prepared using a focused ion beam (FIB) instrument (FEI Helios G5 DualBeam, Thermo Fisher). The process involved the deposition of a silica and platinum protective layer on the original surface, followed by milling the target area to a thickness below 10 nm. High‐resolution TEM (HR‐TEM) images were obtained using a JEM‐2100, JEOL, operating at a 200 kV voltage, while high‐resolution scanning transmission electron microscopy (HR‐STEM) images were obtained using a Titan Cubed Themis, Thermo Fisher, equipped with a field emission gun operating at a 300 kV voltage. The diffraction patterns were analyzed via fast Fourier transform (FFT) using DigitalMicrograph 3 (DM3) software, Gatan. Energy‐dispersive X‐ray spectroscopy (EDS) data were gathered using the Super X annular detector equipped in Themis, and the quantitative analysis of oxygen content was performed using Thermo Scientific Velox software. The electron energy loss spectroscopy (EELS) data were collected using the GIF Continuum System, Gatan. Data processing included the extraction of the target area's spectrum via DM3, followed by the use of Origin software to computine the bandgap through spectral data fitting.

### 4.4 In situ TEM

In situ transmission electron microscopy (TEM) of HfOx RRAM was conducted using a Titan ETEM G2, Thermo Fisher, operating at a 300 kV voltage. The specimen for in situ heating was prepared using the focused ion beam (FIB) technique. The lamella of the device stack was transferred from the substrate to Protochips thermal chips using an EasyLift manipulator. Protochips Fusion software, in conjunction with a Keithley power supply, was employed to heat the chips inside the Titan ETEM Microscope. The heating process commenced at room temperature, with the temperature increased at a rate of 10 °C/s. Concurrently, the microstructural evolution of the HfOx layer was documented via real‐time video in high‐resolution TEM (HR‐TEM) image mode. Any observed drift was corrected using Protochips AXON software.

### 4.5 The DPC‐STEM Characterization

The imaging via differential phase contrast scanning transmission electron microscopy (DPC‐STEM) was performed using a spherical aberration‐corrected STEM (FEI Titan Cubed Themis G2 300) operating at 300 kV. This facilitated the acquisition of high‐resolution STEM images with an approximate spatial resolution of 0.06 nm. The microscope was equipped with a high‐brightness electron gun (X‐FEG with a monochromator), a probe corrector, and an image corrector, in addition to a DCOR+ spherical aberration corrector for the electron probe. A beam semi‐convergence angle of 25 mrad was employed. The collection angle for the DF4 detector was set between 4 and 22 mrad. Prior to data acquisition, the samples were located in the region of interest and left undisturbed overnight to attain stability. Data collection was performed using a rapid scan speed (2 µs/pixel) alongside a high‐resolution (1024 × 1024 pixels), with the aim of further minimizing any residual thermal drift. The differential phase contrast (DPC) data were processed using Avizo software, which enabled the conversion into vector mapping of the atomic electric field.

### 4.6 Quantitative EELS Analysis of Oxygen Vacancy Concentration

The oxygen vacancy concentration was quantitatively determined from the EELS spectra based on the relative intensity of the O‐K edge and Hf‐M_4_,_5_ edges. The procedure was as follows:

#### 4.6.1 Spectral Pre‐Processing

The acquired spectra were first aligned and had their background subtracted using a power‐law model. The signal‐to‐noise ratio was enhanced by principal component analysis (PCA) denoising.

#### 4.6.2 Edge Integral Calculation

The integrated intensities of the O‐K edge (*I_O_*) and Hf‐M_4_,_5_ edges (*I_Hf_*) were calculated over energy windows of 532–560 eV and 1710–1780 eV, respectively, to minimize the influence of fine structure variations.

#### 4.6.3 Atomic Ratio Determination

The O:Hf atomic ratio was obtained using the formula:

$$\frac{N_{O}}{N_{Hf}}=\frac{I_{O}/\sigma_{O}\left(E_{O},\beta \right)}{I_{Hf}/\sigma_{Hf}\left(E_{Hf},\beta \right)}$$

where *N_O_* and *N_Hf_* are the areal densities of O and Hf atoms, *I_O_* and *I_Hf_* are the integrated intensities, and σ_*O*_ and σ_*Hf*_are the calculated partial ionization cross‐sections for the respective edges under the given collection angle β.

#### 4.6.4 Oxygen Vacancy Calculation

The oxygen vacancy concentration *V_O_* (in %) was then derived by comparing the measured O:Hf atomic ratio to the stoichiometric HfO_2_ value (66.6 at.% O):

$$V_{O}=\left(1-\frac{{\left(N_{O}/N_{Hf}\right)}_{measured}}{2}\right)\times 100\%$$

This method yields the oxygen vacancy concentrations ranging from 14.4% to 41.4% as reported in the main text. The uncertainty in the oxygen content (± 3%) was evaluated by combining the standard deviation from four repeated measurements at the same region.

### 4.7 Statistical Analysis

All statistical analyses in this study were performed according to the following protocol:

#### 4.7.1 Pre‐Processing of Data

Electrical data (I–V curves) from the 1K RRAM array were collected without transformation or normalization. Resistance state distributions were evaluated using the cumulative distribution function (CDF) analysis. For microstructural characterizations (HR‐TEM, DPC‐STEM, EELS), data were processed using standard analysis software (DigitalMicrograph, Avizo, Thermo Scientific Velox) as specified in the Methods section. No specific outlier evaluation was performed, as the study focused on reproducible representative device states and atomic‐scale observations.

#### 4.7.2 Data Presentation

Multilevel resistance state electrical characteristics were presented as representative I–V curves. The statistical distribution of eight programmed resistance states across the 1K array is shown using a CDF plot (Figure 1b).

#### 4.7.3 Sample Size (n)

##### 4.7.3.1 Electrical Measurements

Statistical analysis was performed on a total of 1,024 devices (128 × 8 array).

##### 4.7.3.2 Microstructural Analysis

HR‐TEM and DPC‐STEM were conducted on a total of 23 devices, including 7 devices in high resistance state (HRS), 7 devices in medium resistance state (MRS), and 9 devices in low resistance state (LRS). All key observations were reproducible across these samples, as detailed in the main text and figure captions.

#### 4.7.4 Statistical Methods

No formal hypothesis testing (e.g., t‐tests, ANOVA) was employed, as the research goal was to establish qualitative physical correlations through atomic‐scale imaging and representative electrical measurements. The robustness of the findings was supported by consistent and reproducible observation of the relationship between crystallographic orientation, oxygen vacancy distribution, local electric field configuration, and macroscopic conduction mechanism across devices and experimental techniques.

#### 4.7.5 Software used for Statistical Analysis

Origin software (OriginLab) was used for electrical data analysis and CDF plotting. Microstructural data were processed using DigitalMicrograph, Avizo, and Thermo Scientific Velox as described in the Methods section.

## Conflicts of Interest

The authors declare no conflicts of interest.

## Supporting information




**Supporting File1**: advs73453‐sup‐0001‐SuppMat.docx

**Supporting File2**: advs73453‐sup‐0002‐MovieS1.mp4

## Data Availability

The data that support the findings of this study are available from the corresponding author upon reasonable request.
