# Supplementary material for: Atomic‐Scale Mechanisms of Multi‐Resistance States in HfOx‐Based RRAM: Evolution of Atomic Electric Fields and Oxygen Vacancies
Source: Adv Sci (Weinh). 2025 Dec 22;13(10):e18252. doi: 10.1002/advs.202518252 (PMC12915209; doi:10.1002/advs.202518252)
Supplement: Supplementary file 1 — Supporting File1: advs73453‐sup‐0001‐SuppMat.docx [file ADVS-13-e18252-s001.docx]

Supporting Information

Atomic-scale TEM Investigation of Multi-Resistance State Mechanisms in HfOx-based RRAM: Evolution of Atomic Electric Fields and Oxygen Vacancies

*Wen Sun, Yuyan Wang*, Ruofei Hu, Yuyao Lu, Jun Xu, Xinyi Li, Bin Gao, He Qian, Jianshi Tang*, and Huaqiang Wu*

**Table S1.** Spacing calculation of HRS CF in Figure1

|  | **hkl** | **HfO2 (m phase, P21/c)**  **[Å]** | **HRS CF**  **[Å]** | **Error**  **[%]** |
| --- | --- | --- | --- | --- |
| $d_{1}$ | $\overline{1} \overline{1}1$ | 3.131 | 3.185 | 1.7 |
| $d_{2}$ | 0$\overline{2}$0 | 2.575 | 2.591 | 0.6 |
| $d_{3}$ | $1\overline{1} \overline{1}$ | 3.131 | 3.257 | 4.0 |
| $\angle r_{1}r_{2}$ | -- | 52.55 | 50.64 | 3.6 |
| $\angle r_{2}r_{3}$ | -- | 52.55 | 50.6 | 3.7 |

**Table S2.** Spacing calculation of MRS-R4 CF in Figure1

|  | **hkl** | **HfO2 (m phase, P21/c)**  **[Å]** | **MRS CF**  **[Å]** | **Error**  **[%]** |
| --- | --- | --- | --- | --- |
| $d_{1}$ | $\overline{1} \overline{1}$1 | 3.131 | 3.067 | 2.0 |
| $d_{2}$ | $\overline{2}$00 | 2.497 | 2.551 | 2.2 |
| $d_{3}$ | $\overline{1}$1$\overline{1}$ | 2.800 | 2.865 | 2.3 |
| $\angle r_{1}r_{2}$ | -- | 58.2 | 55.71 | 4.3 |
| $\angle r_{2}r_{3}$ | -- | 49.5 | 53.49 | 8.1 |

**Table S3.** Spacing calculation of LRS CF in Figure1

|  | **hkl** | **HfO2 (m phase, P21/c)**  **[Å]** | **LRS CF**  **[Å]** | **Error**  **[%]** |
| --- | --- | --- | --- | --- |
| $d_{1}$ | $\overline{1} \overline{1}$1 | 3.131 | 3.046 | 2.7 |
| $d_{2}$ | $\overline{2}$00 | 2.497 | 2.574 | 3.1 |
| $d_{3}$ | $\overline{1}$1$\overline{1}$ | 2.800 | 3.086 | 10.2 ^a)^ |
| $\angle r_{1}r_{3}$ | -- | 58.2 | 55.35 | 4.9 |
| $\angle r_{2}r_{3}$ | -- | 49.5 | 52.22 | 5.5 |

^a)^ The increase in error may be caused by lattice distortion resulting from the increased oxygen vacancy content in the m phase.

**
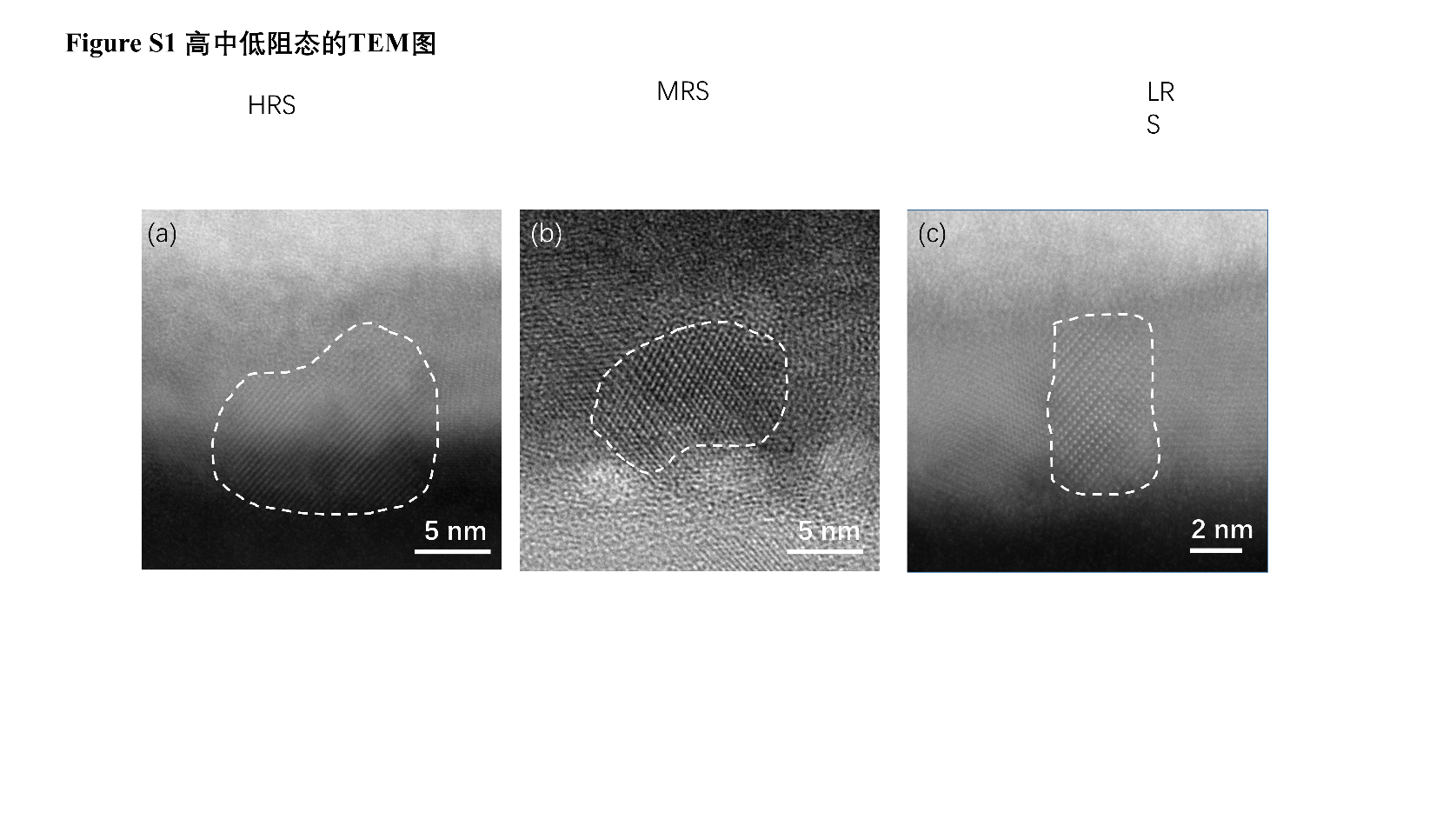
**

**Figure S1**. Representative conductive filament morphologies observed across multiple devices. TEM images confirming the reproducibility of the characteristic filament structures for each resistance state in different devices. (a) A conductive filament (CF) from a separate HRS device, showing the characteristic gap at the HfOₓ/TaOₓ interface (indicated by yellow arrows). (b, c) CFs from separate MRS and LRS devices, respectively, showing the rectangular prism-shaped HfOₓ crystalline structures that bridge the electrodes. The consistent observation of these distinct morphologies across multiple devices underscores the reliability and representativeness of the results presented in Figure 1.

Figure S1 displays typical conductive filaments observed in three different devices under High Resistance State, Medium Resistance State, and Low Resistance State, respectively. These images clearly demonstrate that morphologies of the conduction filaments present in Figure 1.

**
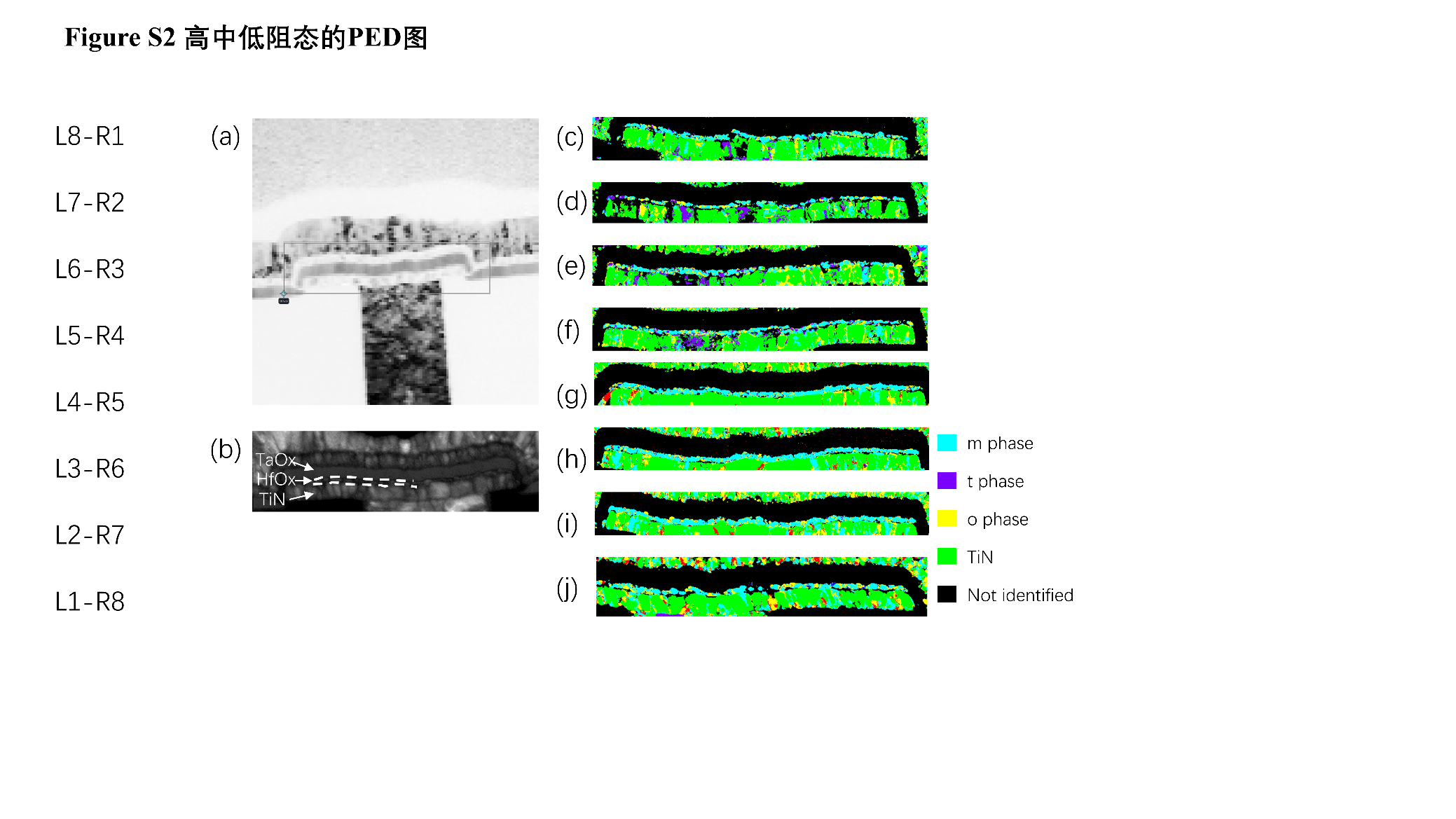
**

**Figure S2** Statistical phase identification of the HfOₓ layer across multiple resistance states via Precession Electron Diffraction (PED). (a) Schematic illustration of the PED data acquisition region within the RRAM device stack. (b) A representative phase-contrast map obtained from PED, color-coded to indicate crystal phases. (c-j) Crystal phase distribution maps for devices programmed to eight distinct resistance states (R1 through R8). In all states, the monoclinic (m-) phase (light blue) is identified as the dominant crystal structure within the HfOₓ layer. These results statistically validate that the m-phase is a universal characteristic of the HfOₓ resistive switching layer in our devices across multi-resistance devices.

To statistically confirm the dominance of the m-phase in the HfO_x_ layer and its association with resistance states, we conducted precession electron diffraction (PED) analysis on devices programmed to eight distinct resistance states (R1-R8) within the programed 1K array. As shown in Figure S2, the PED patterns unambiguously revealed that the m-phase is the predominant crystal phase in the HfO_x_ layer across all tested devices.

**
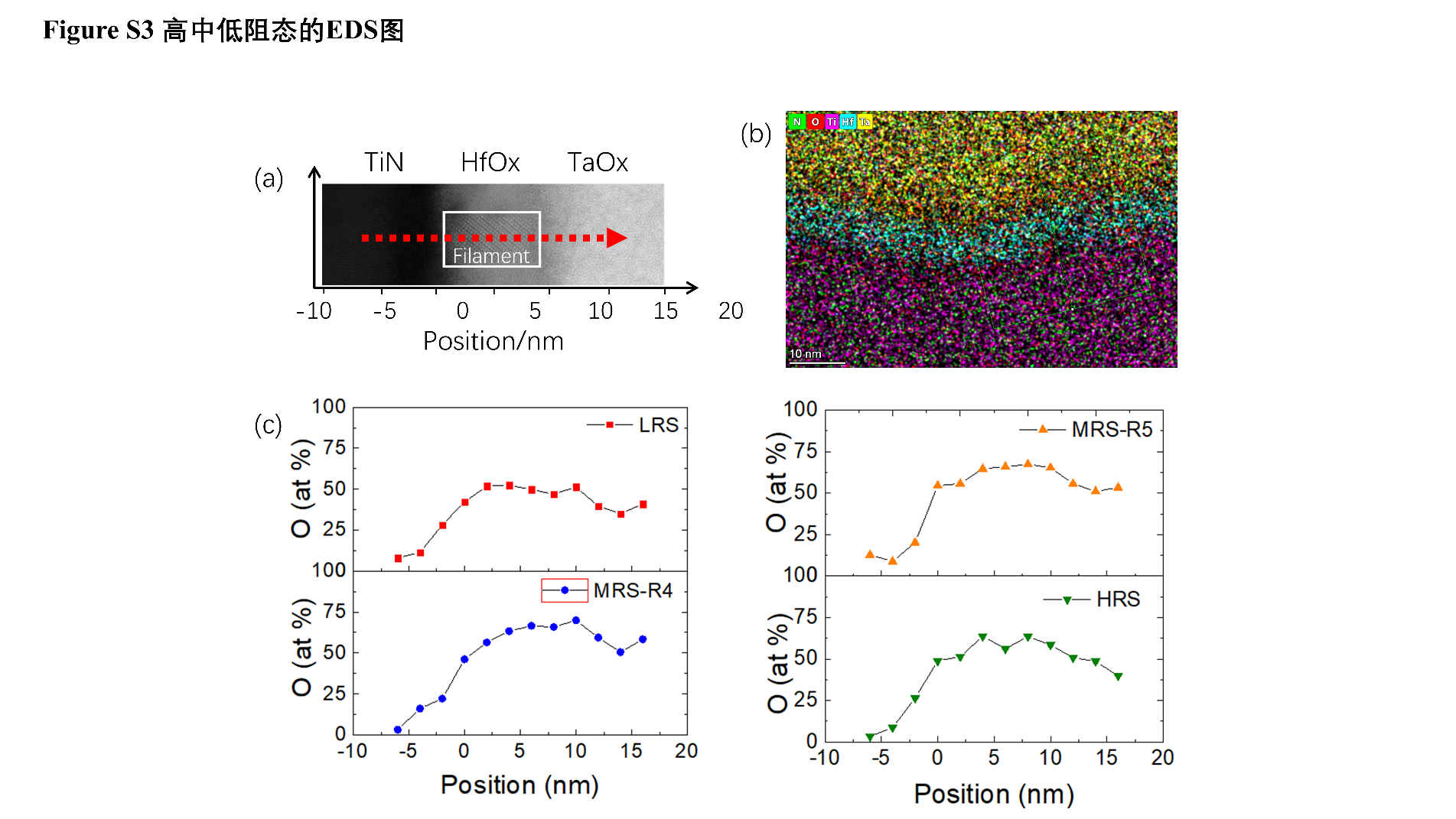
**

**Figure S3** Compositional analysis of conductive filaments across resistance states using EDS in TEM mode. (a) Schematic diagram illustrating the line-scan path along the conductive filament crystal for EDS analysis. (b) Representative elemental mapping (EDS) showing the spatial distribution of Hf (light blue) and O (red) in a TiN/HfO_x_/TaO_x_ stack. (c) Line-scan profiles of oxygen content (atomic %) along the conduction filaments in HRS, MRS, and LRS devices. While the oxygen concentration exhibits a fluctuating trend across all states—consistent with the formation and distribution of oxygen vacancies—the spatial resolution of conventional TEM-EDS precludes atomic-level quantitative accuracy. Nevertheless, the observed fluctuations provide supporting, multi-device evidence for the nanoscale variation in oxygen vacancy concentration associated with different resistance states.

We also employed energy-dispersive X-ray spectroscopy (EDS) in conventional TEM mode to characterize the composition of conductive filament crystals under various resistance states. Figure S3 presents line-scan profiles along the conduction filament direction and elemental mapping. The data indicate that although the distribution of oxygen exhibits nanoscale fluctuations.

**Movie S1**: In situ atomic-scale observation of HfO_x_ RRAM during heating between 515 – 600 ℃. The video is five times faster than actual time.

**Table S4.** The operational temperature of conduction filaments.

| Temperature Range | Measurement/Method | Reference |
| --- | --- | --- |
| 277 °C to 1300 °C (during SET/RESET) | Direct and indirect thermometry (Scanning Thermal Microscopy (SThM), transient pulse thermometry, electrothermal simulations, HRTEM analysis) | ^[1,2]^ |
| 1100 °C to 1300 °C (filament core) | Nanoscale Scanning Thermal Microscopy (SThM) combined with simulations | ^[3]^ |
| > 1327 °C (local) / > 577 °C (heat-affected zone) | Transient thermometry and High-Resolution Transmission Electron Microscopy (HRTEM) | ^[4]^ |
| At least 277 °C (low-power operation, pre-switching) | Indirect methods (specific technique not detailed) | ^[5]^ |
| 627 °C to 1327 °C | Numerical modeling | ^[6]^ |

Multiple studies employing direct and indirect thermometry methods—including scanning thermal microscopy (SThM), transient pulse thermometry, electrothermal simulations, and HRTEM analysis—consistently report local filament temperatures spanning from 277 °C up to 1300 °C during SET and RESET processes.^[1,2]^ For example, direct nanoscale SThM measurements combined with simulations have shown filament core temperatures in HfOₓ-based RRAM devices reaching 1100–1300 °C,^[3]^ while transient thermometry and HRTEM reveal local temperatures surpassing 1327 °C (1600 K) with heat-affected zones above 577 °C (850 K).^[4]^ Even in low-power operation, local filament temperatures of at least 277 °C (550 K) have been confirmed before switching occurs.^[5]^ Numerical modeling similarly predicts filament temperatures between 627 °C and 1327 °C during switching events. ^[6]^

Taken together, these reports confirm that the crystalline phase transitions and m-phase reorientations we observed within the 524.7 °C to 724.4 °C range in our experiments fall well within the established operational temperature window of RRAM conductive filaments. Thus, while our heating protocol was spatially uniform, the phase evolution mechanisms identified—including thermally induced lattice reorientation—are directly relevant to and activated by the highly localized Joule heating present during real device operation.

**Supplementary References**

1. Uenuma, M., Ishikawa, Y. and Uraoka, Y., "Joule heating effect in nonpolar and bipolar resistive random access memory," *Applied Physics Letters* 107, no. 7 (2015): 073503. https://doi.org/10.1063/1.4928661

2. Swoboda, T., Gao, X., Rosário, C. M. M. et al., "Spatially-Resolved Thermometry of Filamentary Nanoscale Hot Spots in TiO_2_ Resistive Random Access Memories to Address Device Variability," *ACS Applied Electronic Materials* 5, no. 9 (2023): 5025-5031. https://doi.org/10.1021/acsaelm.3c00782

3. Deshmukh, S., Rojo, M. M., Yalon, E. et al., "Direct measurement of nanoscale filamentary hot spots in resistive memory devices," *Science Advances* 8, no. 13 (2022): eabk1514. https://doi.org/10.1126/sciadv.abk1514

4. Kwon, J., Sharma, A. A., Chen, C. Y. et al. "Transient thermometry and HRTEM analysis of RRAM thermal dynamics during switching and failure," in *2016 IEEE International Reliability Physics Symposium.* (2016): 7B-3-1-7B-3-5.

5. Yalon, E., Sharma, A. A., Skowronski, M. et al., "Thermometry of Filamentary RRAM Devices," *IEEE Transactions on Electron Devices* 62, no. 9 (2015): 2972-2977. https://doi.org/10.1109/TED.2015.2450760

6. Haris, A., Iris, K., Anel, T., Adnan, M. and Senad, H. "Modelling the generation of Joule heating in defective thin oxide films," in *2016 International Symposium on Industrial Electronics (INDEL).* (2016): 1-4.
